# Unusual Presentation of Phacolytic Glaucoma: Simulating Microbial Keratitis

**DOI:** 10.1155/2011/850919

**Published:** 2011-12-20

**Authors:** Srikant Kumar Sahu, Siddharth Keswarwani, Ruchi Mittal

**Affiliations:** ^1^Cornea and Anterior Segment Service, L V Prasad Eye Institute, Bhubaneswar, Orissa 751024, India; ^2^Miriam Hymen Children's Eye Care Center, L V Prasad Eye Institute, Bhubaneswar, Orissa 751024, India; ^3^Dalmia Ophthalmic Pathologic Services, L V Prasad Eye Institute, Hyderabad, Andra Pradesh 500034, India

## Abstract

The differential diagnoses for phacolytic glaucoma are acute angle closure glaucoma, open angle glaucoma with uveitis, neovascular glaucoma, and glaucoma secondary to trauma. We report an unusual case where the dislocated cataractous lens firmly adherent to the corneal endothelium evoked a cellular reaction similar to phacolytic glaucoma but clinically appeared like a deep corneal abscess. The 73-year-old lady presented with severe photophobia, pain, and redness in the left eye for two months despite being on antibiotics and antifungals. Anterior chamber wash revealed a cataractous lens buried within the infiltrate, which was removed and sent for histopathological examination. Postoperatively she was treated with topical ofloxacin, homatropine, dorzolamide, timolol, and tapering dose of steroids. Histological confirmation of inflammation, histiocytic response, and giant cells around the lens material confirmed the ongoing phacolytic process. Photophobia, pain, and redness subsided following removal of the lens and surrounding cellular reaction. At her last visit, four months after surgery, she was comfortable.

## 1. Introduction

Clinically phacolytic glaucoma presents acutely with corneal edema, cellular exudates in the anterior chamber often with hypopyon, polychromatic hyperrefringent or crystalline particle in the anterior chamber, and hypermature cataract, behind a semidilated pupil with open angle [1]. We report the clinicopathologic correlation of an unusual case where the disrupted hypermature cataract was firmly adherent to the corneal endothelium and surrounded by cellular reaction, mimicking a deep corneal abscess and confirmed by histologic examination.

## 2. Case Report

**Figure 1:**
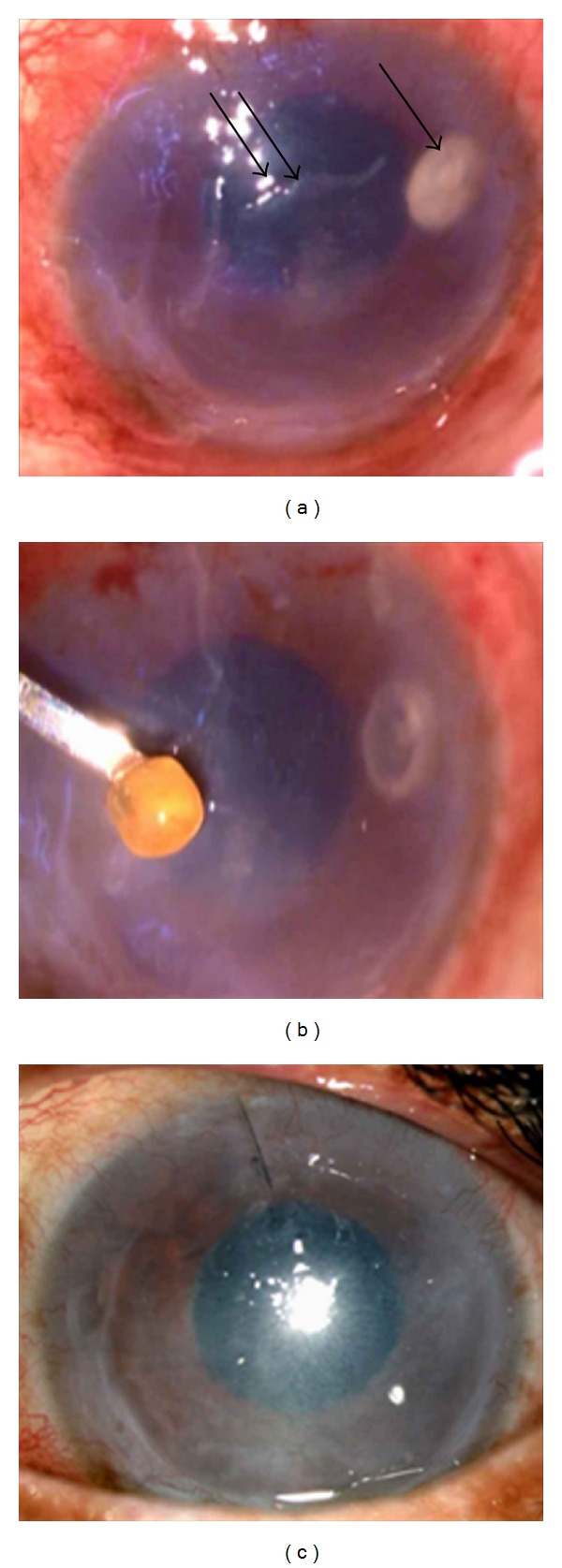
(a) Clinical picture showing infiltrate (arrow) in the corneal stroma with epithelial defect (double arrow) in the surrounding area as seen under microscope. (b) Picture shows yellowish crystalline lens removed from within the infiltrate. (c) Slit lamp picture after one month of surgery showing scarred cornea with a mid-dilated pupil. Note total clearing of the infiltrates.

A 73-year-old lady complained of severe photophobia, pain, and redness in the left eye for two months for which she was prescribed topical antibiotics and antifungals by a local ophthalmologist. She gave history of cataract surgery in the right eye 10 years ago but no history of trauma to either eye. At the time of presentation at our institute, her vision in right eye was 20/70 and in left eye perception of light eye was doubtful. The conjunctiva was congested. There was 2.2 mm × 2.2 mm round deep infiltrate extending to the anterior chamber surrounded by cellular reaction. There was an over lying and few satellite areas of scars surrounding the main lesion (Figure 1(a)). An area of epithelial defect of 2.3 mm × 4.2 mm was present just inferomedial to the infiltrate. The intraocular pressure was high by palpation method. Further assessment could not be done as the patient was very symptomatic. B-scan showed an echo-free vitreous, posterior vitreous detachment, and gross optic nerve head cupping. With a presumptive diagnosis of microbial keratitis, anterior chamber wash was advised. It was done under peribulbar anesthesia with a 2.8 mm limbal incision at 12 o'clock position. With a classical simcoe cannula the infiltrate was aspirated along with a 2 mm × 2 mm of oval, yellow colored mass. Its color, shape, and consistency were similar to the nucleus of a cataractous lens (Figure 1(b)). We also could also identify a separate membrane which was removed.

The smear and culture were negative for any organism. Postoperatively topical ofloxacin (0.3%) eye drop every two hours, homatropine (0.5%) eye drop 3 times per day, and dorzolamide (2%) eye drop and timolol (0.5%) eye drop 2 times per day were started. Tapering dose of steroids was started after a week of the surgery. Four months postoperatively, her symptoms reduced and vision was inaccurate projection of rays. Cornea was scarred (Figure 1(c)). The disc was pale with deep cup.

**Figure 2:**
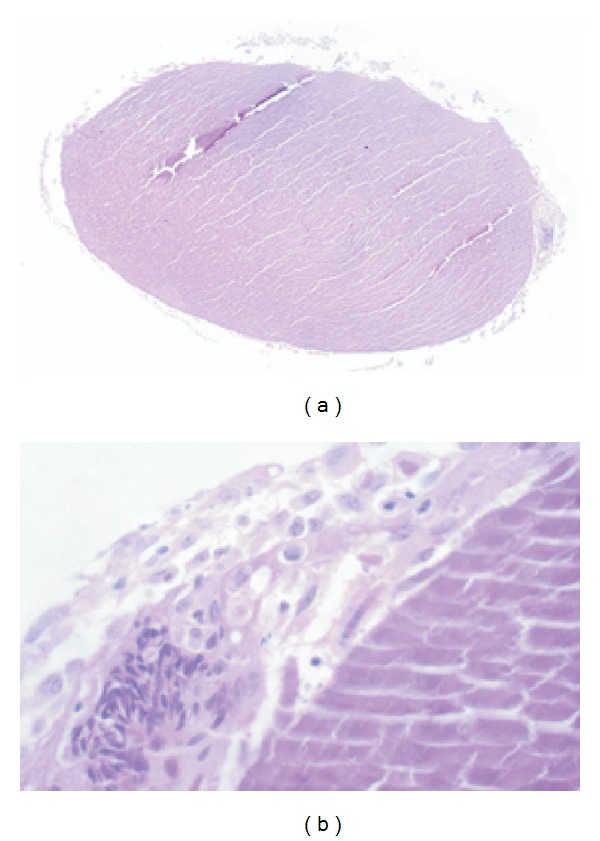
(a) Section shows lens with surrounding inflammatory infiltrates (hematoxylin and eosin, ×100). (b) The higher magnification shows inflammatory cells consisting of polymorphonuclear cells, histiocytes, and engulfed lens matter and multinucleated giant cells (periodic acid Schiff's Stain, ×400).

Histopathologic examination of the excised mass showed the structure of a lens with disrupted fibres, devoid of capsule, and epithelium (Figure 2(a)). The lens was surrounded by inflammatory cells consisting of neutrophils, lymphocytes, and macrophages. There were few multinucleated giant cells engulfing the lenticular fibre (Figure 2(b)).

## 3. Discussion

In the year 1998 Pradhan et al. reported an incidence of 0.42% of phacolytic glaucoma amongst the 27073 of patients detected to have senile cataract [2]. Due to increase in the awareness and availability of cataract surgery, the incidence of phacolytic glaucoma is on the decrease [3]. Acute angle closure glaucoma, open angle glaucoma with uveitis, neovascular glaucoma, and glaucoma secondary to trauma are the conditions which can mimic phacolytic glaucoma [4]. However entrapment of the crystalline lens beneath the cornea along with the surrounding cellular reaction mimicking a microbial keratitis is a novel finding in this case.

Anterior chamber paracentesis has been reported useful in the diagnosis of the etiological agent of infectious keratitis, particularly when the infiltrate is deep stromal or when the presentation is in the form of an endothelial exudates [5–7]. In view of the deep infiltrate extending to the anterior chamber with an overlying scar, we did an anterior chamber tap for etiologic diagnosis. However intraoperatively the crystalline lens was identified within the infiltrate which was adhered to the endothelium. The other membrane which was removed could be an inflammatory exudative membrane; however it was not sent for histopathological evaluation.

Both cellular and humoral immune response has been implicated in the process of phacolytic glaucoma. Phacolytic glaucoma is caused by an obstruction of trabecular meshwork by lens proteins or protein-laden macrophages [3]. Similar to the features seen in AC and vitreous, there was presence of inflammation, histiocytic response, and giant cells seen around the lens material located beneath the cornea thus confirming the ongoing phacolytic process [8, 9].

Though spontaneous dislocation of lens has been reported, we believe that some trivial trauma to the eye with a Morganian cataract could have led to the dislocation of the lens; however a history of trauma could not be elicited [10]. As the patient was very symptomatic an, IOP could not be taken but a gross cupping of the disc (B scan) at the initial visit and a direct view of total cupping on subsequent visits suggests a long standing rise in IOP. Extraction of the lens material has been recommended to relieve the patient of symptoms [8]. Removal of the lenticular material led to relieving of symptoms though the vision could not be revived as the optic nerve damage had advanced.

 In conclusion we report an unusual case of dislocated cataractous lens adherent to the corneal endothelium clinically mimicking microbial keratitis. An anterior chamber wash can safely be used to diagnose and treat such case.
